# Endoscopic Septoplasty—A Narrative Review of Outcomes, Complications and Patient-Reported Score

**DOI:** 10.3390/medicina62010135

**Published:** 2026-01-09

**Authors:** Mihai Alexandru Preda, Zahra Ali Chaloob, Andreea Alexandra Preda, Gabriela Cornelia Musat, Alexandru Iulian Milea, Shirley Tarabichi, Caius Codrut Sarafoleanu

**Affiliations:** 1 Faculty of Medicine, Carol Davila University of Medicine and Pharmacy, 050474 Bucharest, Romania; mihai.preda@umfcd.ro (M.A.P.); andreea-alexandra.nicola@drd.umfc.ro (A.A.P.); mileaalex1993@gmail.com (A.I.M.); shirley.tarabichi@gmail.com (S.T.); csarafoleanu@gmail.com (C.C.S.); 2 ENT Department, ‘Sf. Maria’ Clinical Hospital, 011192 Bucharest, Romania; 3 Department of Internal Medicine and Rheumatology, ‘Sf. Maria’ Clinical Hospital, 011192 Bucharest, Romania

**Keywords:** septoplasty, deviated nasal septum, endoscopic septoplasty, conventional septoplasty, nasal obstruction

## Abstract

*Background*: The main cause of chronic nasal obstruction in ENT practice is represented by the deviated nasal septum. Septoplasty remains the gold standard treatment, performed using either conventional or endoscopic techniques. *Methods*: A narrative review of the literature was conducted using PubMed and Google Scholar for studies published between May 1999 and October 2024. Eligible studies included adult patients (≥16 years) undergoing conventional or endoscopic septoplasty, with at least one reported outcome measure: NOSE, VAS, or SNOT-22 scores; operative time; or complication rates. *Results*: Across multiple clinical studies, both conventional and endoscopic septoplasty provided significant improvements in nasal airflow and symptom relief. Endoscopic septoplasty was consistently associated with superior intraoperative visualization, more precise correction of posterior deformities and isolated septal spurs, and lower rates of intraoperative and postoperative complications. Complication rates were low overall for both approaches. *Conclusions*: Current evidence supports both conventional and endoscopic septoplasty as effective treatments for nasal obstruction due to septal deviation. However, endoscopic septoplasty offers distinct advantages in terms of visualization, operative efficiency, and safety, making it an increasingly preferred technique.

## 1. Introduction

Chronic nasal obstruction is one of the most common complaints encountered in ENT practice. Septoplasty is one of the most frequently undertaken surgical interventions in otorhinolaryngology and represents the standard therapeutic option for correction of a deviated nasal septum.

Surgical management of septal deviation has evolved from classical submucosal resection to conventional septoplasty and extracorporeal techniques. Conventional septoplasty, as described by Killian and Freer, originally [1] is performed under direct visualization, employing a nasal speculum and a headlight. The procedure carries potential complications, which have been extensively reviewed with a focus on strategies for prevention and intervention. More recently, endoscopic septoplasty has gained prominence as a significant advancement, providing enhanced visualization and surgical precision in septal correction [2].

The use of endoscopic techniques for septal correction was first introduced by Lanza and Stammberger in 1991. Subsequently, Lanza et al. provided a detailed description of an endoscopic approach specifically for the management of isolated septal spurs [3].

In addition to the enhanced visualization, it is considered more effective with minimal surgical manipulation and offers the added advantage of simultaneously diagnosing and managing abnormalities of the lateral nasal wall during the same procedure. It allows for a seamless transition between septoplasty and endoscopic sinus surgery when both are performed concurrently, as well as revision procedures. Additionally, endoscopic septoplasty serves as an effective educational tool for surgical training [4].

Several comparative studies have evaluated conventional and endoscopic septoplasty, focusing on improvements in nasal obstruction, operative efficiency, and complication rates. Patient-reported outcome measures, including the Nasal Obstruction Symptom Evaluation (NOSE) score, the Visual Analog Scale (VAS), and the Sino-Nasal Outcome Test (SNOT-22), have become central in the assessment of surgical success.

The purpose of this review is to compare conventional and endoscopic septoplasty with respect to functional outcomes, operative time, patient-reported symptom improvement, and complication rates.

## 2. Material and Methods

This article is a narrative review of the literature. A comprehensive literature search was conducted in PubMed and Google Scholar for studies published from May 1999 to October 2024. Search terms included “septoplasty,” “endoscopic septoplasty,” “conventional septoplasty,” “nasal obstruction,” “NOSE score,” “VAS,” “SNOT-22,” “complications,” and “septal spur.” The reference lists of relevant articles and review papers were also screened to identify additional eligible studies.

Studies were included if they reported clinical outcomes of conventional or endoscopic septoplasty in adult patients (≥16 years), had a sample size of at least 20 patients, and evaluated at least one of the following outcomes: patient-reported measures (NOSE, VAS, or SNOT-22), operative time, anatomical correction, or complication rates. Exclusion criteria were case reports, small series with fewer than 20 patients, pediatric-only studies, non-English publications without translation, and animal or laboratory research.

**Figure 1:**
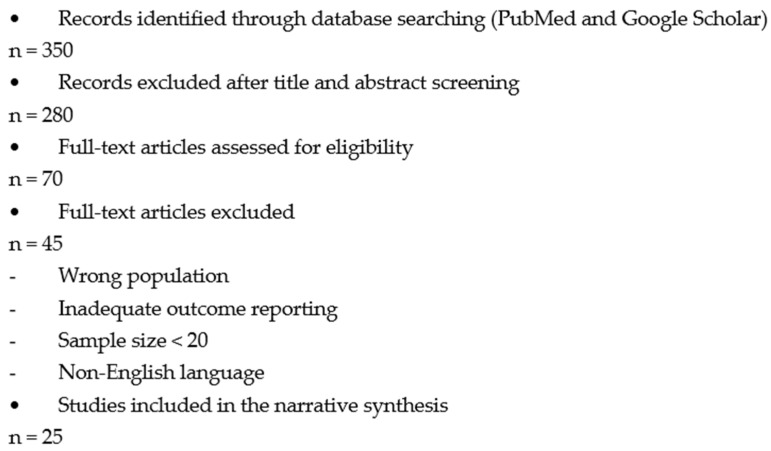
Flow diagram of literature search and study selection.

Extracted data included study design, year of publication, country, sample size, patient demographics, type of septoplasty, operative time, follow-up duration, pre- and postoperative NOSE and VAS scores, complication rates, and key conclusions. Due to heterogeneity in study designs, outcome measures, and follow-up durations, a narrative synthesis of findings was performed. The study selection process is summarized in a flow diagram (Figure 1).

## 3. Classification of Nasal Septum Deviations

Nasal septal deviation may occur because of congenital factors, birth-related trauma, or minor injuries sustained during early childhood. While some deviations are mild and remain clinically silent, more significant deformities can impair nasal airflow and produce symptoms of obstruction [5]. Such deviations often lead to secondary anatomical changes, including hypertrophy of the inferior turbinate on the opposite side. In contrast, the turbinates on the same side as the deviation may demonstrate relative hypoplasia [6].

Lindahl’s classification, proposed in 1954, primarily focused on the aetiology of septal deviations, distinguishing between developmental deviations—typically smooth, “C-shaped” or “S-shaped,” often in the anterior septum—and traumatic deviations, which are usually irregular, angulated, or dislocated. While simple and descriptive, Lindahl’s system does not account for the full spectrum of morphological variations [7]. In contrast, Baumann et al. (2007) developed a more detailed classification that incorporates both the primary deformity and concomitant septal pathologies [8].

One of the earliest and most widely adopted was Cottle’s classification, ref. [9] which divided deviations into five types (I–V) based on the anatomical location of the deformity, from the anterior nasal valve region to the high posterior septum. The nasal anatomy can be divided into five key areas: Area 1 is the nostril, while Area 2 corresponds to the nasal valve. Area 3 lies beneath the bony and cartilaginous framework and is also referred to as the attic. Area 4 includes the anterior portion of the nasal cavity, encompassing the heads of the turbinates and the infundibulum, whereas Area 5 represents the posterior part of the nasal cavity, which contains the tails of the turbinates.

Building on this, Mladina’s classification introduced seven types (I–VII) (Table 1—Mladina classification), providing more detailed descriptions of cartilaginous and bony deformities, often observed using endoscopy [10].

## 4. Technique

### 4.1. Indications

The primary indication for septoplasty is symptomatic nasal airway obstruction. Secondary indications include facilitating access for endoscopic sinus surgery or other transnasal procedures such as transsphenoidal pituitary surgery, correction of anatomic stenosis predisposing to chronic or recurrent rhinosinusitis, optimization of postoperative access for office-based endoscopic examination and debridement, and, less frequently, management of contact point–related headaches. Endoscopic techniques are suitable for correcting most types of septal deviations. Their minimally invasive nature makes them especially effective for addressing isolated posterior deflections, septal spurs, and deviations near septal perforations. This approach is particularly beneficial in revision septoplasty, as it provides superior visualization of tissue planes, allowing for more precise surgical correction [4].

### 4.2. Contraindications

Contraindications to endoscopic septoplasty include situations requiring a septorhinoplasty approach, such as marked caudal deviation or a twisted nose. While the primary benefit of endoscopic surgery is enhanced visualization, this advantage is negated when open approaches to the anterior septum provide adequate exposure using traditional headlight techniques [11].

### 4.3. Surgical Technique

A thorough understanding of the anatomical structures is essential for precise diagnosis, effective treatment planning, and minimizing intraoperative complications [12]. All patients included in the studies received general anesthesia prior to the procedure. Mucosal decongestion was achieved using 0.05% oxymetazoline applied via nasal pledgets, followed by bilateral infiltration of 1% lidocaine with 1:100,000 epinephrine into the subperichondrial and subperiosteal planes [13].

**Figure 2:**
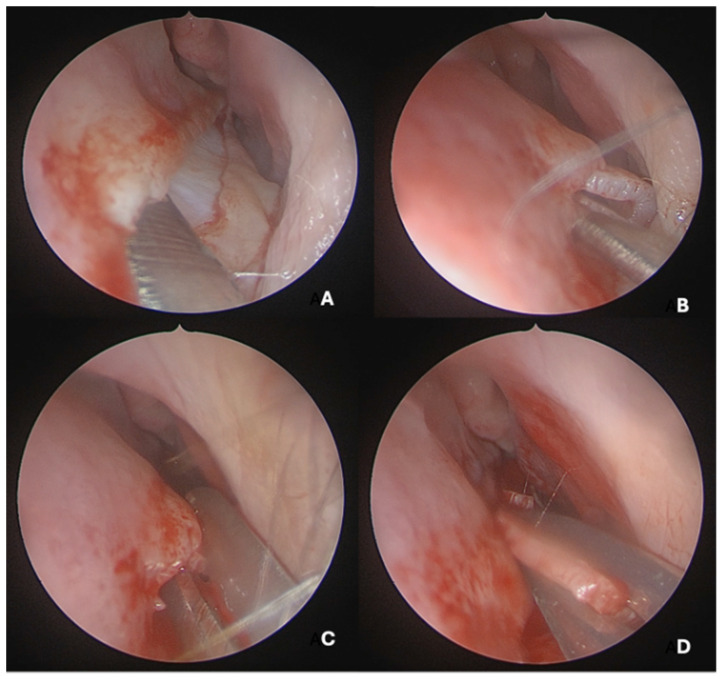
(**A**) Horizontal hemitransfixion incision at the spur apex. (**B**) Flap elevation exposing spur. (**C**) Excising the deviated part of the nasal septum. (**D**) Cartilage resection preserving vomero-chondral junction.

Under endoscopic visualization using a 0° endoscope, the following procedural steps were performed: For most septal deviations, standard Killian or hemitransfixion incisions were utilized (Figure 2). For Mladina types 5 and 6 deformities (corresponding to Cottle’s areas IV and V), a horizontal hemitransfixion incision was placed parallel to the nasal floor at the apex of the septal spur to adequately expose the most deviated segment, ref. [14] whereas more posterior or isolated deformities required a more posterior incision. Following the mucosal incision, mucoperichondrial flaps were elevated with a Cottle elevator, ref. [8] with optimal visualization assisted by a suction Freer elevator. Care was taken when elevating the mucosa over spurs to prevent tears due to thinning [8].

**Figure 3:**
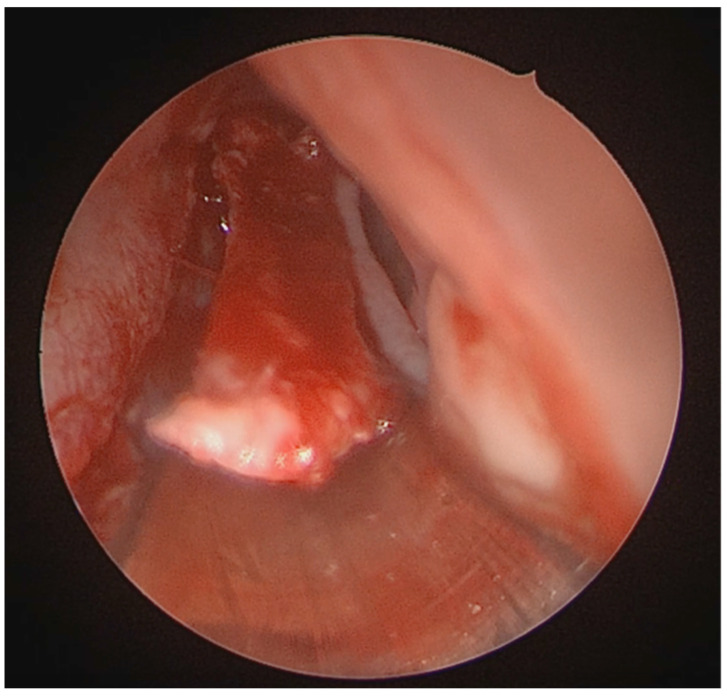
Osteotome positioned at the base of the spur.

**Figure 4:**
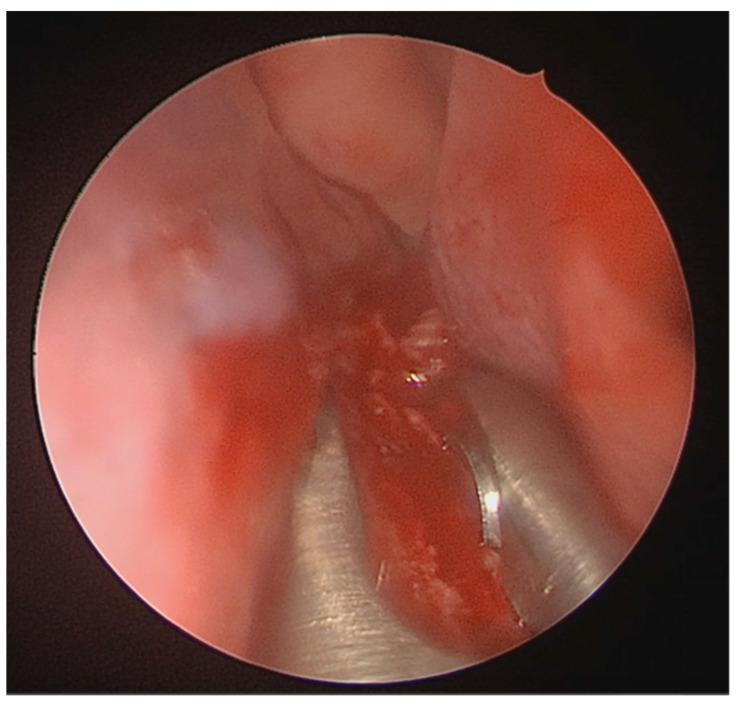
Resection of the deviated nasal spur using Bruenings septal forceps.

A full-thickness vertical incision was made through the cartilage a few millimeters anterior to the point of maximal deviation, which is typically located at the junction of the quadrangular cartilage and the perpendicular plate of the ethmoid [5]. The contralateral mucoperichondrial flap is then elevated in a similar manner. Once the cartilage and bone were separated from the mucosa, the deviated segments were excised using endoscopic instruments including forceps, Brünings punches, and scissors. The deviated portions of cartilage and bone were excised sharply, and the flaps are subsequently dissected inferiorly down to the nasal floor to correct deflections of the vomer. An osteotome was then positioned at the base of the spur to excise the bony protrusion (Figure 3). Deviated bony segments from the vomer or perpendicular plate of the ethmoid were removed as needed (Figure 4). After complete removal of the spur, the mucoperichondrial flaps were restored to their native positions [15]. When flap apposition was satisfactory, suturing was not required; in such cases, the incision line could be covered with a small segment of Gelfilm. Nasal packing and splints were employed only in selected cases [4].

### 4.4. Instrumentation

In most descriptions of endoscopic septoplasty, the instruments used are largely the same as those employed in traditional septoplasty. However, certain tools significantly facilitate endoscopic dissection. The suction Freer elevator is especially useful, as it elevates the mucosal flaps gently while simultaneously aspirating blood to maintain a clear view. An irrigation sheath on the endoscope further improves visualization by keeping the tip clean. When used with a suction elevator or powered instrument, the irrigation fluid is suctioned from the field simultaneously, eliminating the need to remove the endoscope for cleaning and thereby enhancing procedural efficiency. In cases where endoscopic septoplasty is performed in conjunction with endoscopic sinus surgery, the same instrumentation is used, allowing for a seamless transition between procedures. The endoscope enables efficient “back and forth” examination of the submucosal resection and its impact on the nasal airway [16].

### 4.5. Operative Time

Endoscopic septoplasty is generally associated with reduced operative time compared to traditional septoplasty techniques, particularly when a limited approach is used, as suturing is often unnecessary. Studies have reported that limited endoscopic septoplasty can be completed in as little as 12 min on average, whereas classic Cottle septoplasty may require approximately 35 min, nearly three times longer, and submucous resection of the septum takes around 22 min, nearly twice as long. When a Killian or hemitransfixion incision is employed, operative times increase and approach those of conventional septoplasty, reflecting the additional steps required for flap elevation and closure [17].

### 4.6. Advantages

Endoscopic septoplasty offers several advantages [18,19]. The endoscope shortens the surgical time, ref. [20] provides magnification, superior illumination, and enhanced visualization compared with the conventional headlight technique, allowing precise identification of collagen fiber attachments between the perichondrium, periosteum, and underlying cartilage or bone during dissection. When local infiltration is performed under endoscopic guidance, the risk of anesthetic splash into the surgeon’s eyes is effectively avoided. Mucosal injuries can be promptly recognized and carefully managed. Furthermore, unlike the nasal speculum, which may distort normal anatomy, endoscopic visualization preserves a more natural perspective of the nasal cavity [21].

## 5. Results

The included studies, published between 1999 and 2024 (Table 2), comprised randomized controlled trials, prospective cohorts, retrospective series, and meta-analyses. Sample sizes ranged from 34 to 647 patients, with most enrolling between 50 and 120 participants. Patients were predominantly adults in their third and fourth decades of life, and several studies reported a slight male predominance. Follow-up durations varied from 1 to 12 months, with outcomes most assessed at 3–6 months. Across the studies, the primary endpoints were patient-reported outcome measures (NOSE, VAS, SNOT-22) and complication rates, while two recent meta-analyses provided pooled evidence supporting the relative advantages of endoscopic septoplasty [15,22].

Patients in the included studies completed validated questionnaires, most commonly the Nasal Obstruction Symptom Evaluation (NOSE) and the Visual Analog Scale (VAS), and in some cases the Sino-Nasal Outcome Test (SNOT-22) [23]. Preoperatively, scores reflected moderate to severe nasal obstruction across both conventional and endoscopic groups. Postoperatively, all studies demonstrated significant reductions in symptom scores, with greater improvements reported in the endoscopic cohorts. Systematic reviews and meta-analyses confirmed these findings, indicating that while both approaches are effective, endoscopic septoplasty provides superior functional outcomes with fewer complications [18,22].

### 5.1. Patient-Reported Scores

Patient-reported outcomes demonstrated significant improvement following septoplasty, with greater reductions consistently observed in endoscopic groups (Table 3—NOSE scores, Table 4—VAS scores, Table 5—SNOT-22 scores). In a randomized trial of 50 patients, ref. [24] reported mean preoperative NOSE scores of 11.2 ± 2.24 (conventional) and 10.6 ± 1.71 (endoscopic). At one month, these decreased to 7.33 ± 1.50 and 5.00 ± 1.41, and at three months to 6.53 ± 1.25 and 4.40 ± 1.78, with the differences between groups reaching statistical significance (*p* < 0.05) [10]. evaluated 59 patients undergoing endoscopic septoplasty, stratified by Mladina’s classification. Significant reductions were observed across all deformity subtypes (*p* < 0.001), with the greatest improvement in posterior deviations and septal spurs (e.g., type V: 18.1 ± 1.3 preoperatively to 0.8 ± 0.4 at three months) [23]. studied 80 patients and reported high preoperative NOSE item scores, such as 85.8/100 for “trouble breathing” and 82.5/100 for “nasal blockage.” These declined significantly postoperatively (*p* < 0.001). In the same cohort, mean VAS scores improved from 7.6 ± 1.2 to 2.1 ± 0.9 (*p* < 0.001), and SNOT-22 scores from 42.5 ± 7.8 to 12.3 ± 4.6 (*p* < 0.001) at three months, confirming both symptom-specific and broader quality-of-life benefits.

### 5.2. Complications

Among studies providing detailed complication data (Table 6), endoscopic septoplasty demonstrated low overall rates and a safer profile compared to conventional septoplasty [1].

In the largest primary series, Ref. [3] reported complications in 5.3% of 415 patients, most commonly septal hematoma (1.9%) and mucosal tears (1.4%). Residual deviation (1.2%) and infection (0.7%) were less frequent [2]. described mucosal tears and minor bleeding but no major complications in 40 patients, while ref. [4] noted mucosal tears and residual deviation as the most frequent issues, with hematomas rare and no septal perforations observed.

Systematic reviews and meta-analyses provide stronger comparative evidence. Refs. [1,8,25] consistently demonstrated that endoscopic septoplasty was associated with significantly fewer complications than conventional septoplasty, particularly for mucosal tears, septal hematoma, and perforation, while infection rates remained similar.

Collectively, the data confirm that septoplasty is a safe procedure overall, but endoscopic techniques reduce the risk of common complications, likely owing to improved illumination, magnification, and surgical precision.

Findings from systematic reviews and meta-analyses confirm the superiority of endoscopic septoplasty in selected outcomes (Table 7) [8] synthesized 14 studies including more than 1000 patients and found that while both approaches improved symptoms, endoscopic septoplasty yielded greater reductions in NOSE and VAS scores (*p* < 0.05) and was associated with fewer mucosal tears, hematomas, and perforations. In a meta-analysis of 13 randomized controlled trials (735 patients), ref. [25] demonstrated significantly lower postoperative NOSE (*p* < 0.01) and VAS (*p* < 0.01) scores in endoscopic cohorts compared with conventional surgery, together with a reduced risk of complications. Similarly, ref. [1] reviewed 28 studies (2055 patients) and concluded that although both techniques are effective, endoscopic septoplasty provides slightly superior functional outcomes, particularly in posterior deviations and spurs, with these differences reaching statistical significance (*p* < 0.05), and carries a lower overall complication profile.

Overall, all included studies and meta-analyses demonstrated statistically significant postoperative improvements in NOSE, VAS, and SNOT-22 scores, with endoscopic septoplasty consistently providing greater symptom relief and fewer complications compared with the conventional technique (*p* < 0.05 across studies).

## 6. Discussions

The cumulative evidence from comparative studies and meta-analyses indicates that both conventional and endoscopic septoplasty are associated with significant improvements in nasal obstruction and quality of life. Several studies report that endoscopic septoplasty is associated with greater reductions in NOSE and VAS scores and lower reported rates of complications, such as mucosal tears, hematomas, and septal perforations; however, these findings should be interpreted considering the heterogeneous study designs and patient populations included in the literature. The reported advantages of the endoscopic approach are commonly attributed to improved visualization, magnification, and surgical precision, but the magnitude of these differences varies across studies and indications [23].

From a clinical perspective, appropriate patient selection remains crucial [26]. Conventional septoplasty continues to be an effective option for straightforward anterior deviations and remains widely practiced due to its relative simplicity and lower equipment requirements. In contrast, several studies suggest that patients with posterior deviations, isolated septal spurs, revision cases, or those undergoing concurrent sinus surgery may derive particular benefit from an endoscopic approach, where enhanced visualization and targeted dissection are advantageous. Meta-analyses and comparative studies report trends toward improved functional outcomes, shorter operative times, and reduced complication rates in these selected contexts, although the certainty of these findings is limited by methodological heterogeneity [27].

Patient-reported outcome measures provide an important assessment of surgical success. Validated instruments such as the NOSE, VAS, and SNOT-22 scales consistently demonstrate clinically meaningful postoperative improvement following both conventional and endoscopic septoplasty. While some studies report a greater magnitude of improvement with endoscopic techniques, these differences are not uniform across all patient groups or outcome measures and should be interpreted as context-dependent trends rather than definitive comparative effects. These patient-reported outcomes remain central to patient counselling and shared decision-making, supporting individualized surgical planning based on anatomical findings and patient priorities [18,26].

Beyond patient outcomes, the choice of surgical technique has implications for training, education, and institutional practice. Endoscopic septoplasty offers unparalleled visualization of intranasal structures and pathologies, making it an excellent teaching tool for residents and fellows. The enhanced magnification facilitates recognition of anatomical variants and improves surgical precision [28]. However, the approach requires familiarity with endoscopic equipment and spatial orientation, emphasizing the importance of structured training programs and mentorship to minimize the learning curve [29].

Economic considerations are also relevant. Although endoscopic techniques may involve higher initial equipment costs, some studies suggest that these may be offset by shorter operative times, lower complication rates, and the ability to integrate septoplasty with other endoscopic procedures. However, available economic evaluations are limited, and conclusions regarding cost-effectiveness should be interpreted cautiously [16,30].

Emerging technologies, including image-guided navigation, high-definition endoscopes, and angled optics, continue to expand the technical capabilities of endoscopic septal surgery. These developments may further refine patient selection and surgical precision, particularly in complex or revision cases, but their incremental benefit requires further investigation [31].

This narrative review has several limitations. The included studies are heterogeneous with respect to design, sample size, outcome measures, and follow-up duration, and only a limited number of randomized controlled trials are available. Publication bias and selective reporting may also influence the apparent advantages reported for endoscopic techniques. As no formal risk-of-bias assessment or evidence grading was performed, the findings should be interpreted as descriptive and hypothesis-generating rather than definitive.

Future research should prioritize well-designed, multicentred randomized trials with standardized outcome measures and longer follow-up to better define the relative benefits and limitations of each surgical approach.

In conclusion, both conventional and endoscopic septoplasty are effective surgical options for the management of nasal obstruction due to septal deviation. The available literature suggests that endoscopic septoplasty may offer advantages in selected anatomical situations, particularly for posterior deviations, septal spurs, and revision cases; however, current evidence does not support universal superiority or a single preferred technique. Surgical approach should therefore be individualized, considering septal anatomy, associated sinonasal pathology, surgeon expertise, and patient-specific factors.

## 7. Conclusions

Septoplasty remains the standard surgical treatment for nasal obstruction caused by deviated nasal septum and is widely recognized as an effective intervention. The present review indicates that both conventional and endoscopic septoplasty are associated with significant postoperative improvements in patient-reported outcomes, including NOSE, VAS, and SNOT-22 scores. Several randomized trials, cohort studies, and meta-analyses report that endoscopic septoplasty is associated with favourable functional outcomes, particularly in patients with posterior septal deviations and septal spurs; however, the magnitude of these differences varies across studies and should be interpreted in the context of heterogeneous study designs and patient populations.

Beyond symptom relief, several studies describe associations between endoscopic septoplasty and shorter operative times, as well as lower reported rates of intraoperative and postoperative complications. These findings are commonly attributed to improved visualization and targeted dissection afforded by the endoscopic approach, which may facilitate precise anatomical correction and allow for concurrent management of sinonasal pathology. Nevertheless, reported complication rates and operative efficiencies are influenced by surgical experience, case selection, and institutional practice patterns.

Taken together, the available literature suggests that endoscopic septoplasty may offer advantages in selected clinical scenarios, particularly for posterior deviations, septal spurs, revision cases, and procedures combined with endoscopic sinus surgery. However, current evidence does not support universal superiority or a single preferred technique. Both conventional and endoscopic approaches remain effective, and the choice of surgical technique should be individualized based on septal anatomy, associated pathology, surgeon expertise, and patient-specific factors.

## Figures and Tables

**Table 1:** Mladina classification (adapted from Dell’Aversana Orabona et al. [10]).

Type I	Unilateral crest which does not affect the function of the nasal valve. Maintains normal physiological valve angle
Type II	Unilateral vertical septal ridge in the valve region that touches the nasal valve, making the valve angle smaller
Type III	Unilateral vertical ridge that is located more deeply in the nasal cavity, opposite to the middle turbinate’s head
Type IV	A Bilateral deviation consisting of type II on one side and type III on the other
Type V	A nearly horizontal septal spur that extends laterally and deeply into the nasal cavity. The other side of the nasal septum is straight
Type VI	A large unilateral sulcus runs along the caudal-ventral part of the septum, while on the other side is a ridge and causes asymmetry of the nasal cavity
Type VII	A mix of the previous types

**Table 2:** Study characteristics and reported outcomes of endoscopic and conventional septoplasty.

Author, Year	Country	Design	Sample Size (n)	Sex (M/F)	Mean Age (yrs)	Follow-Up	Outcomes Reported
Hwang et al., 1999 [4]	USA	Retrospective	111	Not stated	34	6 m	Indications, complications
Gupta, 2005 [2]	India	Prospective	50	30/20	28	6 m	Complications
Kulkarni et al., 2015 [3]	India	Retrospective	415	236/179	31	12 m	Complications, revision rate
Dell’Aversana Orabona et al., 2018 [10]	Italy	Prospective	59	32/27	29	6 m	NOSE scores by deformity
Shukla et al., 2020 [23]	India	Prospective	80	44/36	27	3 m	NOSE, VAS, SNOT-22
Garzaro et al., 2019 [15]	Italy	Prospective	647	370/277	36	12 m	Complications, outcomes
Bhardwaj et al., 2023 [24]	India	RCT	50 (25Conv/25Endo)	28/22	31	3 m	NOSE (Conv vs. Endo)
Besharah et al., 2023 [25]	China	Meta-analysis	735 (13 RCT)	Mixed	Variable	Variable	NOSE, VAS, complications
Trimartani et al., 2023 [1]	Indonesia	Meta-analysis	2.055 (28 studies)	Mixed	Variable	Variable	NOSE, VAS, complications

**Table 3:** NOSE scores.

Study	n	Pre-op NOSE (Conventional)	Pre-op NOSE (Endoscopic)	Post-op NOSE (Conventional)	Post-op NOSE (ENDOSCOPIC)	Follow-Up
Bhardwaj, 2023 [24]	50	11.2 ± 2.24	10.6 ± 1.71	7.33 ± 1.50 (1 mo) 6.53 ± 1.25 (3 mo)	5.00 ± 1.41 (1 mo) 4.40 ± 1.78 (3 mo)	1 & 3 months
Dell’Aversana Orabona, 2018 [10]	59	-	Type I: 14.1 ± 1.2 → 12.3 ± 1.7 (3 mo), 12.0 ± 1.9 (6 mo) Type II: 16.0 ± 1.0 → 15.6 ± 1.0, 15.5 ± 1.0 Type III: 15.0 ± 0.8 → 14.5 ± 0.9, 14.1 ± 0.6 Type IV: 17.8 ± 0.5 → 11.0 ± 3.7, 10.2 ± 3.3 Type V: 18.1 ± 1.3 → 0.8 ± 0.4, 0.7 ± 0.4 Type VI: 17.6 ± 1.4 → 7.9 ± 7.7, 7.7 ± 6.7 Type VII: 16.6 ± 1.2 → 15.3 ± 0.6, 15.0 ± 1.0	-	See (Endoscopic) column	3 & 6 months
Shukla et al., 2020 [23]	80	-	NOSE items: “trouble breathing” 85.8/100; “nasal blockage” 82.5/100	-	Significant reduction	1 & 3 months

**Table 4:** VAS scores.

Study	n	Pre-op VAS (Conventional)	Pre-op VAS (Endoscopic)	Post-op VAS (Conventional)	Post-op VAS (Endoscopic)	Follow-Up
Shukla et al., 2020 [23]	80		7.6 ± 1.2		2.1 ± 0.9	3 months

**Table 5:** SNOT-22 scores.

Study	n	Pre-op SNOT-22 (Conventional)	Pre-op SNOT-22 (Endoscopic)	Post-op SNOT-22 (Conventional)	Post-op SNOT-22 (Endoscopic)	Follow-Up
Shukla et al., 2020 [23]	80	-	42.5 ± 7.8	-	12.3 ± 4.6	3 months

**Table 6:** Reported complications following endoscopic and conventional septoplasty in selected clinical studies.

Author, Year	Sample Size (n)	Technique (s)	Complication Type	Details
Kulkarni et al., 2015 [3]	415	Endoscopic	Septal hematoma	8 cases (1.9%)
			Mucosal tears	6 cases (1.4%)
			Residual deviation	5 cases (1.2%)
			Infection	3 cases (0.7%)
			Total	22 cases (5.3% overall)
Gupta, 2005 [2]	40	Endoscopic	Mucosal tears	Few cases (not quantified)
			Minor bleeding	Occasional; controlled intraoperatively
			Major complications	None reported
Hwang et al., 1999 [4]	111	Endoscopic	Mucosal tears	Reported, frequency not specified
			Residual deviation	Reported, occasional
			Hematoma	Rare
			Septal perforation	None reported
Besharah et al., 2023 [25]	—(meta-analysis of RCTs)	Conventional vs. Endoscopic	Pooled complications	Endoscopic group had significantly fewer mucosal tears, hematomas, and perforations; infection rates similar
Trimartani et al., 2023 [1]	—(PRISMA systematic review)	Conventional vs. Endoscopic	Pooled complications	Endoscopic septoplasty safer overall; reduced hematoma, mucosal tears, and perforation rates
Hong et al., 2016 [8]	>1000 pooled (14 studies)	Conventional vs. Endoscopic	Septal hematoma	Lower in endoscopic group
			Mucosal tears	Lower in endoscopic group
			Septal perforation	Lower in endoscopic group
			Infection	Comparable between groups

**Table 7:** Comparative postoperative outcomes of endoscopic and conventional septoplasty.

Author, Year	Studies/Patients	Post-op NOSE (Conventional)	Post-op NOSE (Endoscopic)	Post-op VAS (Conventional)	Post-op VAS (Endoscopic)	Key Findings
Hong et al., 2016 [8]	14 studies; >1000 pts	Improved	Greater improvement	Improved	Greater improvement	Endoscopic superior in symptom reduction; fewer mucosal tears, hematomas, perforations
Besharah et al., [25]	13 RCTs; 735 pts	Improved	Significantly lower NOSE	Improved	Significantly lower VAS	Endoscopic superior in nasal obstruction relief and associated with fewer complications
Trimartani et al., 2023 [1]	28 studies; 2055 pts	Improved	Slightly superior, especially for posterior deviations and spurs	Improved	Improved	Both techniques effective; Endoscopic associated with lower complication rates

## Data Availability

The original contributions presented in this study are included in the article. Further inquiries can be directed to the corresponding authors.

## Abbreviations

The following abbreviations are used in this manuscript:

VAS	Visual Analogue Scale score
SNOT-22	Sino-Nasal Outcome Test-22
NOSE	Nasal Obstruction Symptom Evaluation
ENT	Ear, Nose, Throat
